# Valorising Whey: From Environmental Burden to Bio-Based Production of Value-Added Compounds and Food Ingredients

**DOI:** 10.3390/foods14213646

**Published:** 2025-10-25

**Authors:** Hiba Selmi, Ester Presutto, Giuseppe Spano, Vittorio Capozzi, Mariagiovanna Fragasso

**Affiliations:** 1 Department of Agriculture, Food, Natural Science, and Engineering (DAFNE), University of Foggia, 71122 Foggia, Italy; ester.presutto@unifg.it (E.P.); mariagiovanna.fragasso@unifg.it (M.F.); 2 Institute of Sciences of Food Production, National Research Council (CNR) of Italy, c/o CS-DAT, Via Michele Protano, 71122 Foggia, Italy; vittorio.capozzi@cnr.it

**Keywords:** dairy industry, whey, microbial processing, materials, fermentation, challenges

## Abstract

Cheese manufacturing generates large volumes of whey with high biochemical and chemical oxygen demand, historically treated as waste. Yet, whey is rich in lactose, proteins, and minerals that can be fractionated and upgraded into foods and bio-based products. During cheese production, 80% to 90% of the total volume is discarded as whey, which can cause severe pollution. However, milk by-products can be a natural source of high-value-added compounds and a cost-effective substrate for microbial growth and metabolites production. The current review focuses on cheese whey as a key milk by-product, highlighting its generation and composition, the challenges associated with its production, methods for fractionating whey to recover bioactive compounds, its applications in functional food development, the barriers to its broader use in the food sector, and its potential as a substrate for producing value-added compounds. Particularly, the focus was on the recent solutions to use cheese whey as a primary material for microbial fermentation and enzymatic processes, producing a diverse range of chemicals and products for applications in the pharmaceutical, food, and biotechnology industries. This review contributes to defining a framework for reducing the environmental impacts of whey through its application in designing foods and generating biomaterials.

## 1. Introduction

Dairy processing contributes substantively to global nutrition and economies by converting raw milk into yoghurt, cheese, butter, and powders [1]. The constant increase in the global population resulted in a proportional rise in milk production, reaching nearly 970 million tons worldwide in 2023. The production is expected to slightly increase by 0.5–0.9% annually [2]. Thus, a significant amount of residual materials is generated, amounting to millions of tons each year [3]. Side-streams grow proportionally, generating substantial wastewater, losses, and by-products, most notably whey [4]. These residual materials refer to (i) the discarded wastewater used for the cleaning of the equipment, (ii) food loss generated from raw milk and other dairy products due to their expiration dates or improper production, transportation, and/or storage process, and (iii) milk by-products [5]. Milk by-products include secondary products released during milk processing that can be edible, non-edible, or even considered waste. Examples of milk by-products include milk residues, skimmed milk, cream, and whey [6]. However, managing milk wastes and by-products represents a significant challenge for agri-food companies, especially the management of whey, because it requires treatments before disposal [7]. Indeed, whey contains a high concentration of organic load with a high biological oxygen demand (BOD) and a high chemical oxygen demand (COD), estimated to 35–45 kg/m^3^ and 60–70 kg/m^3^, respectively [8], and the incorrect discharge of whey can reduce the oxygen dissolved in water, and therefore, disturbance of the aquatic ecosystem [9]. From 10 litres of milk utilised to produce 1–2 kg of cheese, about 8–9 litres of whey is generated [10]. Most commonly, whey was released into sewage systems or water bodies like lakes, rivers, and oceans, discharged for animal feeding, or sprayed onto farmland as fertiliser. However, in all cases, whey leads to groundwater contamination [11]. Along these lines, it is necessary to develop alternative methodologies to reduce their environmental impacts. According to the European Directives 91/271/EEC and 97/771/ECC, wastewater derived from the cheese industry should undergo special treatment before direct discharge [12]. Numerous industrial pre-treatments were proposed in order to reduce the organic load and other solid pollutants of wastewater, including electrocoagulation, electro-oxidation, solid-phase micro-extractions, aerobic treatments using Jet loop bioreactors, and membrane systems [13,14,15]. However, these technologies involve complex and long processes, with high costs [16]. On the flip side, whey is rich in valuable nutrients, including lactose, proteins, and lipids; therefore, it can be considered as a natural source of high-value-added compounds and a cost-effective substrate for microbial growth and metabolites production [17].

Cheese whey is a pale green-coloured transparent liquid with a pH value ranging between 4.5 and 6.0 [18]. Its composition depends on the type of milk and the type of manufactured cheese. Recently, whey has been utilised as a substrate for various microbial and enzymatic processes to produce important and diverse chemicals with applications in the pharmaceutical, food, and biotechnology industries [19,20]. The fermentation of cheese whey by indigenous microbes through batch dark fermentation resulted in the conversion of carbohydrates to lactic acid (23 mmol/g total initial organic carbon), which was further transformed into short-chain fatty acids and hydrogen [21]. Whey-lactose fraction can be utilised as a fermentation medium for *Trametes versicolor* to produce edible films with antioxidants [22]. Likewise, whey can serve as the sole carbon source for the strain *Bacillus flexus* Azu-A2, enabling it to produce a biopolymer alternative to plastics [23]. Further studies have investigated the production of hydrogen from cheese whey using the photo-fermentative bacterium *Rhodopseudomonas* sp. strain BR0Y6, achieving a productivity of 87 mL/L/d [24].

In addition to molecule production, foods developed using cheese whey were stable, rich in bioactive metabolites, and had an acceptable sensory profile [25]. In this context, Trejo-Flores and colleagues suggested using whey as a base to formulate high-quality ice cream, introducing mango seeds as a raw source of carbohydrates and fats, since cheese whey is a low-fat by-product [26]. Indeed, the incorporation of postbiotic-enriched whey into yoghurt showed the highest radical scavenging activity (18.71% inhibition) and the best overall acceptability compared to the other yoghurt samples [27]. In line with these findings, it was reported that probiotics have the potential to produce functional cheese whey-based beverages [28,29]. The preparation of whey-based beverages with fruit juices enhanced their antimicrobial and antioxidant activities [30]. Whey obtained from ricotta cheese was used to prepare a probiotic sports beverage, fermented by ABT YO-MIXÔ 205 mixture and fortified with algae powder [31]. The co-production of postbiotics, including linoleic acid, exopolysaccharides, and bacteriocins, was assessed by *Bifidobacterium lactis* BB12 in medium-supplemented cheese whey [32]. Furthermore, the hydrolysis of cheese whey by *Streptococcus thermophilus* has generated anti-hypertensive lactotripeptides, including valine-proline-proline and isoleucine-proline-proline, with concentrations of 12.8 mg/L and 0.56 mg/L, respectively [33].

From this perspective, converting cheese whey into valuable products will help reduce pollution and generate sustainable products. The current review paper aims to provide a comprehensive overview of whey, structured in a logical research chronology: starting from its generation, compositional differences based on milk source, potential contaminants, biochemical approaches for whey fractionation, and finally, its valorisation in food applications and compound production. Challenges related to its introduction into the food industry are further investigated. The contribution of the current article includes combining microbiological and biochemical aspects to reduce the environmental impact of whey by turning it into valuable compounds.

## 2. Generation of Whey by the Dairy Industry and Its Composition

Approximately 82% and 14% of the world’s fresh milk are from cows and buffaloes, respectively, while the rest comes from goats, sheep, and camels [34]. During the production of caseins and cheeses, the remaining liquid after milk coagulation and casein removal is discarded as milk by-products, called “whey”. However, whey contains approximately 50% of the milk’s nutritional composition [35]. Whey can be classified into two primary categories: sweet whey and acid whey, based on the type of cheese produced [36].

Sweet whey is produced during cheese-making products (semi-hard and hard cheeses) and other casein products (yoghurt, butter, ice cream) through casein coagulation using rennet or casein-coagulating enzymes (proteolytic enzymes) [37]. In contrast, the processing of acid whey involves casein precipitation through lactic fermentation or the addition of organic acids, such as citric, acetic, or lactic acids [38]. The majority of fresh cheeses (cottage cheese, ricotta, Greek yoghurt) are made using this type of whey, and, as its name indicates, the pH value of acid whey ranges from 4.5 to 5.8 [39]. Acid whey contains a lower amount of proteins and lactose than sweet whey, which might be explained by the acid coagulation occurring near the isoelectric pH of casein, leading to the precipitation of more milk proteins. Therefore, acid whey is rich in ash, minerals, and lactic acids [40].

Generally, whey is composed of water (93–95%), soluble proteins, lactose, minerals, and vitamins. Whey proteins comprise globular proteins, such as β-lactoglobulin and α-lactalbumin, which represent 50% and 25% of the whey protein fraction, respectively, as well as minor components including immunoglobulins, lactoferrin, casein, and serum albumin. These peptides contain essential amino acids, such as branched-chain and sulfur amino acids, which can impact the properties of the protein fraction of the whey [41]. In addition to whey proteins, caseins are phosphoproteins that can bind strongly to polyvalent cations such as calcium, resulting in neutralisation and precipitation. Four main caseins, namely αS1-, αS2-, β-, and κ- caseins, in which αS1-casein is the most abundant in milk [42]. β-Lactoglobulin is a retinol-binding protein belonging to the superfamily of lipocalin; It can bind to a variety of molecules and hydrophobic ligands, including cholesterol, vitamin D2, and iron ions [43]. Regarding α-Lactalbumin, it is a calcium-binding metalloprotein and a regulatory molecule for the enzymatic system of β-galactosyl transferase, responsible for lactose synthesis [44]. Whey proteins can contain immunoglobulins and traces of lactoferrin, accounting for about 6% and <1% of whey protein, respectively. Lactose (milk sugar) is a disaccharide, which by hydrolysis yields glucose and galactose monomers [45]. It has a low caloric value, a low glycaemic index, and it is less sweet than sucrose and glucose. Its favourable characteristics are plasticity, compressibility and light flavour [46].

The nutritional composition of whey cheese was assessed, and the results are presented in Table 1. It was found that proteins and minerals are available in whey cheese in considerable amounts, ranging from 10.4 to 11.4 g/100 g and 3.6–6.9 mg of calcium per gram, respectively. Similarly, Domiati cheese whey, an Egyptian soft white pickled cheese, contains 65.9 g/L lactose, 3.8 g/L protein, and 2.4 g/L fat [47]. In contrast, goat whey provides a higher protein and fat content, with approximately 14.4–16.7 g/100 g and 6.9–7.9 g/100 g, respectively [48]. The origin of the milk might explain this difference; generally, goat milk is richer in fats (4.3 g/100 g) than cow milk (3.7 g/100 g) [49], and sheep whey is the richest in lactose (5.3 g/100 g) [37]. Additionally, the composition of milk components might vary due to several factors, including environmental conditions, animal nutrition, stage of lactation, and animal breed [50]. Comparative studies have affirmed that mid-lactation whey cheeses exhibit higher concentrations of lactose than those obtained from early and late lactation whey [51]. No difference was observed in protein content from cheese whey produced in cheese factories or local farms, while individual protein analysis revealed that cheese whey collected from farms had higher concentrations of lactoferrin and caprine serum albumin than cheese whey from dairy factories [52]. Indeed, a Norwegian team has evaluated the compositional variation in whey obtained from two cheese types (Cheddar cheese and Dutch cheese). Results showed that whey collected from Dutch-type cheese production in the middle or northern regions during the spring season contained the highest concentration of proteins. In addition, it was found that protein concentration is primarily affected by the season of production; the concentration of β-lactoglobulin was significantly higher during winter and summer compared to spring, while α-lactalbumin was richer during summer compared to spring and autumn [53]. Further studies consider that the influence of the season is through the feeding regimes, which, in consequence, can affect milk characteristics [54,55]. McGuinness and colleagues investigated the influence of diet and stage of lactation on the rheological and sensory properties of Cheddar cheeses. Results showed a clear difference in the colour and texture of Cheddar cheese. Consumers rated cheeses produced from perennial ryegrass and partial mixed ration cow diets as more intense than those from total mixed ration cow diets in flavour, saltiness, and aftertaste [56]. Generally, acid whey contains 4.2–4.9% of lactose, 0.55–0.75% of proteins, 0.8% of ash, and about 0.04% of lipids [57]. Acid whey is rich in minerals and lactic acid because less of the calcium is trapped within the curd, and higher calcium content in acid whey causes calcium lactate formation [58].

## 3. Challenges Associated with Whey Production

**Figure 1:**
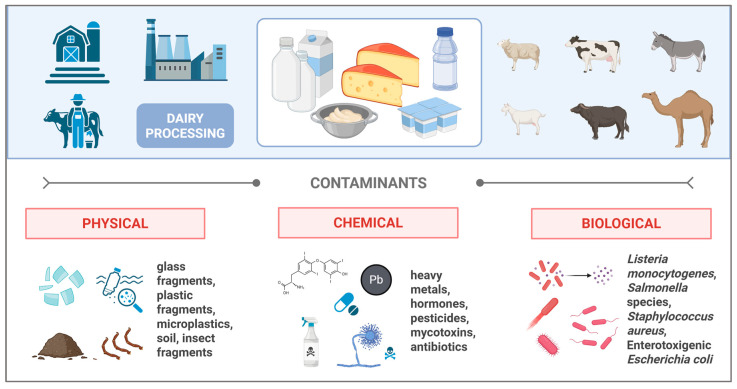
Sources and types of contamination during milk processing. Created in BioRender https://BioRender.com/qa3xir3.

As already mentioned, the quality and the composition of whey are greatly influenced by the quality of the milk and the coagulation process (type, temperature, duration). These processes are among the most important factors affecting (i) the microbial quality of the whey [66]. For example, uncleaned and/or unsanitized milking equipment can be a source of pathogenic microbes belonging to the genera *Bacillus*, *Geobacillus*, *Anoxybacillus*, *Brevibacillus*, *Paenibacillus*, and *Clostridium*. Additionally, cross-contamination can occur due to negligent handling or the use of infected tools during processing or transportation (Figure 1). *Staphylococcus aureus*, *Salmonella enterica*, *Bacillus cereus*, and *Listeria monocytogenes* were previously reported in milk products [67,68,69]. In 2018, as part of USDA’s National Animal Health Monitoring System, Sonnier and colleagues assessed the provenance of zoonotic pathogens in 234 dairy operations. *Salmonella enterica* was detected in 18% of total dairy farms, including some strains that were found to be resistant to antimicrobials. *Listeria* spp. were present in 19.9% of operations, while *Listeria monocytogenes* was isolated from 3% of operations [70]. Recently, another study affirmed the occurrence of *Salmonella* spp. in milk and dairy products [71]. Likewise, spore-forming *Bacillus cereus* is a common contaminant of dairy products due to its high distribution in the environment. The prevalence of this microbe in raw milk can range from 3.8% to 100%, and in pasteurised milk, its occurrence ranges from 2% to 65.3% [72].

In Italy, the occurrence of *Bacillus cereus* in dairy products ranges between 18.3% and 33% [73,74,75]. Seventy-four samples were collected from food and non-food contact surfaces in a medium-scale dairy industry in order to understand the contamination route of *Bacillus cereus* strains. Data obtained from environmental samples showed a similar distribution of *Bacillus cereus* in the different areas dedicated to cheese production (4/37 samples for the production area, 2/28 samples for the brining/ripening area, 2/4 samples for the packaging area). Furthermore, the presence of *Bacillus cereus* was detected in various locations, including the water hose, the floor of the production area, the trolley wheels in the packaging area, and all surfaces in contact with the floor. The final product was *Bacillus cereus*-free, which can be attributed to the good practices and proper management during milk processing [76]. Another challenge to face is biofilm-forming bacteria, which can form microbial biofilms on equipment surfaces. These microbial biofilms can act as a protective structure for pathogenic microbes. Due to its high water activity, whey is conducive to microbial growth and toxin production. Spore-forming bacteria can persist in complex environmental conditions, including high or low pressure, biocides, UV radiation, and extreme temperatures. To increase food safety, a recent study demonstrated the efficacy of combining thermal treatment with the introduction of polyvalent phage LPEK22 to control the growth of multidrug-resistant bacteria, including *Escherichia coli* and *Salmonella enterica* [77]. In order to inactivate microorganisms and enzymes in milk, non-thermal technologies with low energy input have been proposed. For example, high-power/high-intensity ultrasounds, often in conjunction with mild heating (thermosonication), were found to be efficient in reducing microbes in milk [78]. In addition, a protective culture containing *Lacticaseibacillus* strains was proposed by Pires and colleagues to prolong the shelf life of concentrated bovine cheese whey by reducing the content of yeasts and moulds after 28 days of storage [79]. Sameli and colleagues suggested the application of crude lactic acid bacteria bacteriocin (addition of 5% *v*/*w* enterocin crude extract) as a preventive method to inhibit spoilage microbiota in whey cheese [80].

(ii) Chemical contaminants can be found in whey, as they can enter the dairy supply chain at various stages of milk processing. In fact, dairy production follows several stages: it begins with feed production, followed by raw milk production/collection, and then further processing at the farm, and finally, at dairy companies [81]. Significant chemical hazards include pesticides, antibiotics, hormones, plastics, mycotoxins, and heavy metals can be detected in dairy products. A study analysed 79 cheese samples collected from small farms in Croatia for the presence of pesticide residues and heavy metals. Out of 509 pesticides, only two samples contained the active substance piperonyl butoxide, as detected by LC-MS/MS. Concentrations were between 0.043 and 0.038 mg/kg, while all the others were below the limit of quantification < 0.01 mg/kg. Cd, Cr, Mn, Ni, and Pb were found in the range of <0.005–0.012 mg/kg, <0.02–0.84 mg/kg, 0.031–1.128 mg/kg, <0.03–0.67 mg/kg, and <0.01–0.12 mg/kg, respectively [82]. Lead was detected in all milk, whey, and cheese samples, exceeding the maximum permissible lead limit in milk set by European Union legislation, which ranges from 0.04 to 0.13 mg/kg. In whey, the mean concentrations ranged between 0.04 mg/kg to 1.36 mg/kg, and were listed in decreasing order as follows: Fe > Zn > As > Se > Sr = Ni > Al > Cu > Cr = Li > Pb > Mn = Co > Ba > Sb > Mo [83]. In addition, the detection of organochlorine pesticides in dairy products was assessed using Gas chromatography with electron-capture detection. Results showed a reduction in organochlorine pesticide content after fermentation, with processing factors ranging between 0.42 and 0.64 in yoghurt and 2.37 and 4.93 in cheeses, respectively [84].

Rafoxanide, a drug used to treat liver fluke, a disease that can seriously impact animal health and productivity, was assessed in raw milk and dairy products. Findings showed that rafoxanide was concentrated sixfold in cheese compared to raw milk (2070 μg/kg vs. 349 μg/kg), but four times lower in whey (75 μg/kg). In butter, rafoxanide residues were more than 14 times higher than in the starting milk, with levels of 5468 μg/kg and 376 μg/kg, respectively [85]. In line with these findings, it has been widely reported that microbes, especially probiotic lactic bacteria, can reduce pesticide residues in foodstuffs through hydrolysis or adsorption [86]. Therefore, combining microbial processes with techniques such as ultrasonic irradiation can intensify and enhance/accelerate pesticide degradation [87,88]. The ozone treatment for 60 min has reduced aflatoxin M1 in whey concentrate by 10% [89]. Recently, it was reported that microalgae can eliminate contaminants from milk by-products by using them as nutrients [90,91].

Further study assessed the presence of antibiotic residues in raw bovine milk using liquid chromatography-tandem mass high-resolution spectrometry (LC-HRMS). Findings revealed that all detected antimicrobial residues were below the maximum residue limits (MRLs). Additionally, Lincomycin residues were observed in 30 samples, with a frequency of 11.8%. Three samples showed oxytetracycline at concentrations of 15.05, 0.82, and 1.59 ppb, and two samples showed cefapirin and spiramycin at trace levels [92]. The transfer of antibiotics from raw goat milk to the whey obtained after rennet coagulation was evaluated using microbial inhibitor tests. It was found that β-lactam antibiotics are the most released from curd and transferred to the whey [93]. In line with these findings, Escobar Gianni and her colleagues fortified raw milk with seven antibiotics, including ampicillin, penicillin G, cloxacillin, dicloxacillin, cephalexin, tetracycline, and oxytetracycline. After the processing of cheeses and whey powder, ampicillin and penicillin G residues were detected in whey at concentrations similar to those added to raw milk. Cephalexin was mostly distributed in whey, ranging from 82% to 96%, with the antibiotic presenting the highest concentration in whey powder [94]. The distribution of cefquinome in different dairy products was investigated. The antibiotic primarily migrated with the skimmed milk, rather than the cream, during butter manufacture, but was equally distributed in the curd and whey during cheese manufacture [95]. Heat treatment for 5 min at 100 °C was examined to degrade veterinary antibiotics, including aminoglycosides, tetracycline and trimethoprim, which were reduced to about 30% of the starting amount [96]. In addition, antibiotic residues can be removed chemically from milk using ozonation in a vortex reactor. It was reported that the reduction of ceftiofur hydrochloride, sulfamonomethoxine sodium, marbofloxacin and oxytetracycline in milk [97].

(iii) Microplastics have been widely detected in the environment, food supply chain, and even in tissues and human organs. The size limit of this plastic debris ranges from 1 µm to 5 mm [98]. In raw milk, it was reported that more than 200 small-sized microplastics were found in 100 mL of cow’s milk [99]. The average abundance of microplastics in commercialised yoghurt and buttermilk was between 0.63 and 0.76 item/mL and 0.5 and 0.7 item/mL, respectively, with sizes ranging from 1 mm to 5 mm [100]. In addition, skim milk powder can be contaminated by 29 different types of polymers, including polypropylene, polyethene, polystyrene, and polyethene terephthalate. Microplastics were found in three different forms: fibre, sphere, and irregular fragments [101]. A Chinese study revealed the contamination of flavoured yoghurt by microplastics utilising microscopic Raman imaging. The identified materials were primarily polystyrene, polypropylene, and polyethene, constituting approximately 20%, 3%, and 1%, respectively [102]. While current research has indicated the presence of microplastics in milk and milk products, they are likely prevalent in whey, a milk by-product. However, to date, no published study has confirmed this.

## 4. Fractionation of Whey for the Recovery of Bioactive Compounds

The dry matter of cheese whey retains most of the lactose (66–77%), globular proteins (8–15%), along with mineral salts (7–15%). It contains a mixture of proteins that need to be isolated and purified to exploit their functional and nutritional characteristics fully. In general, whey contains 20% of the total proteins of milk and about 50% of milk solids. Indeed, the fractionation of whey components facilitates the valorisation and the use of proteins, lactose, and minerals separately. The protein part can be converted into emulsifiers, gelling agents, antimicrobials, enzymatic cofactors, or preservatives in functional food [103,104,105,106]. Whey lactose can be valorised into oligosaccharides used as prebiotics, in functional food, flavour and texture enhancer, and/or drug carrier and coating agent for tablets in pharmaceuticals [45,107,108].

**Figure 2:**
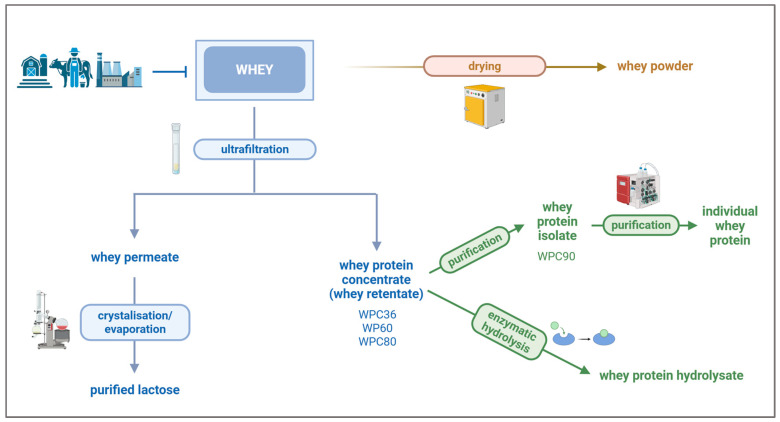
Overview of possible fractionation of whey and the resulting product. Created in BioRender. https://BioRender.com/jmn9vvd.

The separation of lactose from whey is well established, where ultrafiltration and nanofiltration are utilised to produce a concentrated lactose solution, which will be further evaporated, crystallised, and/or dried, in order to obtain lactose powder. At the same time, the retained whey proteins on the membrane, during the ultrafiltration process during lactose recuperation, are further accumulated to produce whey protein concentrate [109]. Generally, whey protein concentrates (WPC) are constituted by up to 80% protein (*w*/*w*). The most common whey protein concentrates include WPC34, WPC60, and WPC80, which contain 34%, 60%, and 80% of proteins, respectively (Figure 2). In addition, the composition of whey protein concentrate 34% is similar to the composition of skim milk [8]. WPC34, WPC60, and WPC80 are rich in essential amino acids with unique functional and nutraceutical properties. The removal of extra fats from WPC80 increases the protein content to 90% (WPC90) and the generation of whey protein isolate (WPI) (Table 2) [8]. The separation of proteins depends on several physical and chemical properties, including size, charge, solubility, and environmental conditions (e.g., pH, temperature, ions, conductivity, and concentration) [110].

In a commercial scale, the separation process must be simple, rapid, and maintain the product’s quality with high yield and purity. Over the last few decades, several fractionation technologies based on (*i*) membrane separation, (*ii*) chromatography, (*iii*) enzyme hydrolysis, and precipitation have been developed [111]. (*i*) Membrane filtration processes have been widely applied in dairy manufacturing, serving as permeable barriers to separate whey constituents based on their size, electrical charge, or concentration [112,113]. Microfiltration can be utilised as a pre-treatment step for fat and bacteria removal prior to whey protein concentration. Later, ultrafiltration membranes with a molecular weight cut-off (10–20 kDa) are typically used to fractionate proteins from lactose and minerals. The retentate/concentrate can be used as a starting material for the isolation of specific whey proteins using ultrafiltration membrane with molecular weight cut-off ranging from 10 to 300 kDa [113]. Nanofiltration step can be further applied for demineralisation and deacidification [46]. Recently, membranes used for ultrafiltration have been modified by incorporating charged functional groups to increase their efficacy and selectivity. For example, α-lactalbumin and β-lactoglobulin proteins have very similar sizes, but different isoelectric points; therefore, they can be separated using charged membranes [114]. In another study, a polyethersulfone-based membrane was developed by incorporating Pluronic F127 and carbon nanotubes with single- and multi-walled dimensions as additives to fractionate α-lactalbumin from whey protein [115]. Electrodialysis is another separation technique widely used in the dairy industry in order to remove salts [116]. It is based on the use of filtration membranes that are not permeable to molecules and ions greater than 500 Da [117]; in other words, lactose and proteins are rejected. Electrodialysis can be combined with ultrafiltration to achieve enhanced selectivity, where mobility is manipulated by both size and electrical charge [109,118]. (ii) Chromatography is an alternative technology applied in whey protein separation, usually resulting in higher protein purity than proteins obtained by the membrane approach [119]. Serum albumin can be fractionated from cheese whey using affinity chromatography with immobilised llama antibody fragments [120]. The use of a peptide ligand attached to a resin allowed production of an α-lactalbumin-rich fraction with a purity of 90.6% [121]. Additionally, cation-exchange using a 5 mL SP Sepharose FF column can be employed to separate α-lactalbumin and β-lactoglobulin from whey concentrate powders, with a recovery of 78% of total β-lactoglobulin at 95% purity [122]. An innovative protein separation strategy, based on coupling high hydrostatic pressure with acidification of whey, has been proposed by Marciniak and colleagues to fractionate α-lactalbumin and β-lactoglobulin from cheese whey [123]. Several researchers have been applying the (iii) enzymatic hydrolysis process to produce bioactive peptides using proteases [124]. It is the most established method for releasing peptides from whey protein; the enzymes involved are generally derived from bacteria, fungi or plants. Enzymes, including alcalase, flavourzyme, papain, α-chymotrypsin and bromelain, are widely employed in whey protein hydrolysis [125]. Acidic enzymes like protease A and protease M showed a hydrolysis selectivity toward α-lactalbumin [126], while trypsin targeted β-lactoglobulin [127]. Whey protein concentrate can be hydrolysed using a protease mixture of subtilisin and chymotrypsin, therefore improving whey protein hydrolysate wettability and solubility [128]. The proteolytic system of *Streptococcus thermophilus* and *Lactobacillus delbrueckii* subsp. *bulgaricus* can be involved in whey protein hydrolysis [129].

**Table 2:** The chemical composition of whey fractions, as per recent findings. n.d.: No Data.

	Total Solids (% *w*/*w*)	Protein (% *w*/*w*)	Fat (% *w*/*w*)	Lactose (% *w*/*w*)	Ash (% *w*/*w*)	Reference
**Whey**	6.3–30.9	0.7–14.2	0.1–12	3.7–4.9	0.6–0.7	[8,130]
**Second whey**	6.0–7.0	0.7–0.9	0.1–0.8	4.2–5.0	n.d.	[10]
**Sweet whey**	6.1–6.6	0.78–1.7	0.05–0.1	4.2–5.3	0.5–0.7	[8,131,132]
**Acid whey**	6.2–8.7	0.5–1.8	0.07–0.4	3.9–5.1	0.7–1.8	[8,132,133,134]
**Whey powder**	n.d.	11.0–14.5	1.0–1.5	63.0–75.0	8.2–8.8	[8]
**Demineralised whey**	n.d.	11.0–15.0	0.5–1.8	70.0–80.0	1.0–7.0	[8]
**Whey permeate**	5.2–6.0	0.1–0.4	<0.01	4.7	0.52	[135,136,137]
**WPC34**	n.d.	34.0–36.0	3.0–4.5	47.7–52.0	6.1–8.0	[8,138]
**WPC60**	n.d.	56.3–62.0	1.0–9.0	25.0–30.0	4.0–6.0	[8,138]
**WPC80**	n.d.	76.0–82.0	4.0–8.3	4.0–8.0	0.9–4.0	[8,138]
**WPI**	n.d.	90.0–92.0	<1.5	0.5–1.0	2.0–8.0	[8,131,139]

n.d.: No Data.

It was found that physical pre-treatments, like ultrasound and high hydrostatic pressure, can enhance enzymatic efficacy and increase the degree of hydrolysis [140,141,142]. Further alternatives for the hydrolysis of biopolymers and proteins include subcritical water hydrolysis, a method that utilises water at a temperature between 100 and 374 °C and pressures below 22 MPa to adopt properties similar to those of non-polar solvents such as hexane [143]. Lactose in cheese whey can be hydrolysed enzymatically by β-galactosidase, yielding a net energy gain of 9166.7 KJ [144]. The bioconversion of lactose enhanced substrate availability for methane production, resulting in a reduction in soluble chemical oxygen demand to around 24.6% and a 75.8% reduction in volatile fatty acid concentration [145].

## 5. The Use of Whey in the Production of Functional Food

In recent decades, findings have revealed the possibility of employing whey in the production of new foods. Cheese whey can be reused to produce fresh milk products, such as ricotta, brunost, and whey butter, or utilised as a food ingredient for protein powder [146,147]. In this sense, several approaches have been developed to convert whey into a resource of valuable compounds and to minimise the disposal problem.

### 5.1. The Production of Prebiotics

Lactose and protein, as main compounds of whey, were recognised to exhibit numerous nutritional, functional, and physiological features, which make them potentially utilised for versatile applications. Indeed, the use of whey lactose can be an interesting alternative substrate for the synthesis of prebiotics. Numerous studies have suggested that lactose derived from whey can serve as a potential source for the production of lactulose, galacto-oligosaccharides, lactitol, lactosucrose, lactobionic acid, and tagatose [45]. It was found that administering galacto-oligosaccharides can improve stool frequency in adults who self-report constipation. 11 g of galacto-oligosaccharides significantly increased faecal *Bifidobacterium* and *Anaerostipes hadrus* [148]. Lactitol relieves constipation by regulating gut microbiota and improving gastrointestinal transit in Sprague Dawley rats [149]. The prebiotic potential of lactulose, galactooligosaccharides, and lactosucrose on the proliferation of probiotics and their potential to enhance short-chain fatty acids production were investigated by Wu and colleagues. Findings revealed that lactosucrose most effectively promotes the proliferation of *Lacticaseibacillus* and *Bifidobacterium* strains, accompanied by an increased lactic acid yield reaching 4.02 mg/mL of *Lacticaseibacillus casei* CS-773 in anaerobic conditions [150]. D-tagatose is a monosaccharide that can be transformed into short-chain fatty acids by colonic bacteria, and it is generally recognised as safe by the Food and Drug Administration [151]. In the colon, lactulose restores and maintains good gut health by increasing beneficial bacterial flora over pathogenic bacteria and enhancing mineral absorption [152]. Ammonium carbonate can be used as an alkaline catalyst to produce lactulose from whey. At high temperature (96.82 °C) and at a concentration of 0.76% ammonium carbonate, the production yield of lactulose reached its maximum of about 29.6% [153]. To increase lactulose synthesis from cheese whey and fructose substrate, β-galactosidase was immobilised on chitosan activated with glutaraldehyde. The immobilisation has increased the productivity from 24.6 g/L/h to 31.5 g/L/h in treated whey (precipitation of whey proteins) [154]. Another study assessed the hydrolysis of lactose whey by immobilisation of *Kluyveromyces lactis* NRRL Y1564 β-galactosidase. The hydrolysis of lactose was successfully performed with a level of lactose hydrolysis of 86% and lactulose production of 17.32 g/L [155]. In an enzyme membrane reactor system, the biosynthesis exhibited a high conversion rate after 10 batches, using cellobiose 2-epimerase, with a production of about 84.5 g/L of lactulose [156]. Further study aimed to isomerise lactose into lactulose using electro-activation technology with the chemical isomerisation method using KOH as a catalyst. The highest lactulose yield of 32% was achieved at a 900 mA current intensity for 60 min with a 7% whey solution [157]. A cost-effective whey medium was developed to optimise β-galactosidase production by *Lactobacillus bulgaricus* L3. The conversion of whey lactose into galacto-oligosaccharides by incubating crude enzymes with 200 g/L whey (about 160 g/L lactose) resulted in a production yield of 44.7% [158]. Likewise, Limnaios and his colleagues succeeded in producing galacto-oligosaccharides enzymatically from whey lactose. The team employed three β-galactosidases obtained from *Kluyveromyces lactis*, *Aspergillus oryzae*, and *Thermothielavioides terrestris*, and the maximum galacto-oligosaccharide yield ranged from 23.7% to 25.7% [159]. In addition, Orrego and Klotz-Ceberio were capable of producing galacto-oligosaccharides from the remaining whey lactose after protein concentration. The commercial β-galactosidase showed galacto-oligosaccharides a maximum yield of 74% (g galacto-oligosaccharides/g lactose) with a lactose utilisation of 63%, in 30 min of reaction [160]. Milk whey and whey permeate were utilised to produce galacto-oligosaccharides using immobilised *Bacillus circulans* β-galactosidase. In the batch process, the immobilised enzyme exhibited excellent operational stability at 40 °C, being able to hydrolyse 91% lactose after 17 cycles of reuse. The maximum concentration of galacto-oligosaccharides achieved, using Milk whey and whey permeate, was 159.4 g/L and 168.8 g/L, respectively [161]. Recent findings demonstrated the ability of whey protein isolate conjugated to stachyose to modulate the digestibility of proteins and carbohydrates by stimulating sustained-release effect in the gastrointestinal tract and repair intestinal barrier [162,163]. In vivo study revealed that the uptake of whey protein supplements enhanced the gut microbiota composition and increased the species *Lactococcus lactis* and *Bacteroides vulgatus* in mice [164]. Likewise, Nielsen and colleagues suggested that whey protein concentrate improve the growth rate and the immune system of preterm pigs [165].

### 5.2. The Production of Food Functional Ingredients

Increasing evidence supports the potential introduction of whey in the food sector as a food additive to enhance functional properties, including nutritional value and rheological properties [166,167]. Thus, the incorporation of whey cheese in Queso fresco cheese was evaluated. It was found that adding 6% or 8% (*w*/*w*) whey protein to cheeses increased the production yield by 16% and 18%, respectively. The resulting cheeses were softer and exhibited cohesiveness similar to commercial Queso fresco cheese [168]. Likewise, cheese whey was utilised as a foaming agent to prepare ricotta cheese. An increase in foam stability was detected, with the best results from a combination of 1% xanthan gum and 3% albumin powder in whole whey (Whey 100: Milk 0) and whole milk (Milk 100: Whey 0) ricotta cheese [169]. Further study revealed the ability to utilise acid whey of Petit Suisse-type cheese for the formulation of fermented milk, substituting water [57]. The physicochemical, microbiological, and sensory properties confirmed the feasibility of using goat and sheep liquid whey concentrates in the dairy industry to formulate frozen yoghurts [170]. Additionally, the use of whey protein concentrate (from processed cheese whey) in the manufacture of ovine ice cream was evaluated. Ice creams prepared with whey protein concentrates (65% or 80% protein) had lower water activity (aw) and lower overrun (36–46%) than ice cream prepared with bovine skimmed milk, and the flavour score of all ice creams was similar [171]. In addition, the incorporation of whey protein isolates into ice creams influenced the rheological properties, showing an increase in viscosity indicators, a uniform distribution of air bubbles, and good stability of purity and colour intensity during storage [172]. Whey protein was further added as a functional ingredient to formulate ice cream. The by-product used was obtained from the recovery of milk cheese-making residues [173].

Recent papers also highlight the suitability of whey for microbial fermentation using lactic acid bacteria and/or yeasts [174,175]. In fact, fermentation is one of the most cost-effective ways to preserve food, enhance sensory properties, and improve nutritional value. Lactic acid bacteria were primarily used for the production of functional foods based on whey cheese, including *Lactobacillus acidophilus*, *Lactocaseibacillus casei*, *Lacticaseibacillus rhamnosus*, and *Limosilactobacillus reuteri* [176,177,178]. To enhance the sensory properties of acid whey, researchers have proposed introducing lactic fermentation through the probiotics *Lactobacillus acidophilus* LA-5 or *Bifidobacterium animalis* ssp. *lactis* BB-12. The production involves a combination of pasteurised acid whey with ultra-high temperature milk, condensed milk, or skim milk powder. Beverages containing *Lactobacillus acidophilus* showed higher acidity, which increased during storage, whereas the acidity of samples containing *Bifidobacterium animalis* was more stable. It was found that the best sensory properties were in a beverage made from whey, milk, condensed milk, and *Lactobacillus acidophilus* [179]. Further study investigated the production of a fermented probiotic beverage based on whey (25%) and pineapple juice (75%), with *Lactobacillus acidophilus* LA-5 as the starter culture. The probiotic whey-pineapple beverage could be preserved for 56 days at 4 °C with satisfactory acceptability and shelf-life, and the viable probiotic cell count was 4.2 Log_10_ CFU/mL [180]. Likewise, combining whey protein concentrate and kiwi powder can create an interesting mixture for a functional probiotic beverage. Lactic bacteria, including *Streptococcus salivarius* subsp. *thermophilus*, *Lactobacillus acidophilus*, and *Bifidobacterium animalis* subsp. *lactis* were used for fermentation. The obtained whey beverage exhibited higher phenolic compounds and amino acid contents, along with an interesting antioxidant activity, compared to the control [181]. Furthermore, *Pediococcus pentosaceus* ENM104 and *Lactiplantibacillus plantarum* SPS109 were found to be suitable as a microbial mixture for producing fermented whey beverage [182]. Further studies have developed a mango-flavoured yoghurt beverage using acid whey obtained from Greek yoghurt. Findings indicated that the addition of 25–35% acid whey is most preferred by consumers based on sensorial and chemical characteristics [183]. Cunha and her colleagues suggested using *Enterococcus malodoratus*, a high gamma-aminobutyric acid-producing strain isolated from cheese, to naturally enrich sweet whey beverages with gamma-aminobutyric acid. After 14 days, gamma-aminobutyric acid content reached 300 mg/100 mL, which is comparable to the content of commercially marketed GABA supplements [184].

A recent study investigated the ability of lactic acid bacteria-fermented whey to extend the shelf life of bread against fungal contamination after 7 days. Whey was fermented with *Lactiplantibacillus plantarum* 5L1 strain, and then added to the dough. Results showed an increase in organic acid and phenolic compound content after fermentation. Bread with 5% concentration of fermented whey had visible mould only after 7 days, compared to the control, where mould appeared after only 4 days [185]. Furthermore, whey powder fermented by *Lactiplantibacillus plantarum* strains exhibited antifungal activity against several fungi, and the addition of the supernatant of fermented whey-based medium to doughs improved the shelf life of the bread [186]. The addition of 20% whey protein concentrate to the dough increased protein digestibility and enhanced the sensory features of the bread, including a higher browning index and increased hardness [187]. Likewise, it was reported that substituting flour with whey protein isolate prolonged dough development time, enhanced dough stability, and improved farinographic quality [188]. Further study proposed incorporating acid whey into tomato sauces. The optimal water substitution by acid whey was 70% *w*/*w*, resulting in increased lactose and calcium content, without causing any off-flavours and affecting their shelf-life [189].

## 6. Challenges in Introducing Whey in the Food Industry

Despite the benefits, whey is generally associated with several key challenges, including rapid microbial spoilage, scaling issues with processing equipment, flavour defects resulting from high mineral content, sensitivity of microbial cultures to fermentation conditions, and phase separation of the final product. The sour and salty taste of acid whey is the primary challenge in incorporating it into whey-based products [190]. Additionally, the acid whey composition can impact the odour, flavour, and aftertaste of the final product. Acid whey is richer in minerals and lactic acid than sweet whey; therefore, demineralisation and deacidification are required [58]. In baked goods, high mineral and high lactic acid content can impair evaporation and act as water structure promoters, accelerating the “caking” process and reducing flowability [191]. Additionally, the introduction of whey powder can result in a sticky product due to its high lactose content [192]. In cheese, high lactose contents impact the gelation process, reducing gel hardness and resulting in a weak gel [193]. To overcome the issue, lactose crystallisation before drying was proposed [194]. Further major challenge when producing protein hydrolysates is the release of hydrophobic and bitter peptides [127]. LC-TOF-MS/MS was used to characterise the bitter peptides in order to remove them [195]. Some amino acids, including l-histidine, l-valine, l-arginine, l-isoleucine, and l-phenylalanine, were found to exhibit a bitter taste [196]. Therefore, several physicochemical and biological approaches have been developed to prevent and reduce the formation of bitterness compounds in foodstuffs [197]. Physico-chemical methods, including masking of bitter tastes, Millard reaction, encapsulation, and enzymatic hydrolysis, are currently applied at the industrial level. The masking method is based on the addition of agents to regulate the sensory characteristics. The most common masking agents are β-cyclodextrin, gelatin, malic acid, modified starch, and citric acid [198,199]. To mask the bitter taste in whey protein hydrolysate beverages, Leksrisompong and colleagues suggested the use of sucralose, fructose, sucrose, adenosine 5′ monophosphate (5′AMP), or adenosine 5′ monophosphate disodium (5′AMP Na(2)) [200]. The Maillard reaction refers to a non-enzymatic process involving a complex chemical interaction between the nucleophilic groups of peptides and the carbonyl groups of sugars [201]. The Maillard reactions can influence not only the sensory properties of the food, but also its nutritional quality and its stability [202]. Recently, Bu and colleagues succeeded in removing phenylalanine from whey protein hydrolysates through enzymatic hydrolysis by endopeptidases and exopeptidases, followed by resin adsorption [203]. The encapsulation by the spray drying method is frequently employed to remove unwanted flavours and to enhance the stability of bioactive compounds [204]. The biological debittering methods include the enzymatic hydrolysis by selective peptidases derived from microbes, the removal of ammonia from peptides by hydrolysing amide group by enzymes, or plastein reaction [197]. Interestingly, the fermentation of whey with probiotics or/and the addition of fruit juices did not significantly affect the sensory properties and did not cause undesirable taste [30,178].

## 7. The Use of Whey in the Production of Compounds

### 7.1. Organic Acids

The utilisation of whey as a cheap, renewable substrate to generate value-added products is investigated. The production of (i) lactic acid from whey permeate was assessed by Sahoo and Jayaraman using *Lactobacillus delbrueckii* and engineered *Lactococcus lactis* strains [205]. In monoculture with *Lactobacillus delbrueckii*, the highest yield of lactic acid production was 0.48 g/g when the initial lactose concentration was 30 g/L. Whereas *Lactococcus lactis* TSG1 yielded 0.67 g/g and 0.44 g/g of lactic acid from lactose and galactose, respectively. A co-culture batch process of *Lactobacillus delbrueckii* and recombinant *Lactococcus lactis* achieved an enhanced production yield of 0.90 g/g from whey permeate (lactose). In batch production, *Lactobacillus paracasei* cultured in deproteinized whey supplemented with yeast extract (4 g/L) reached a yield of 1.05 g/g of lactic acid [206]. It was reported that the ability of *Lactiplantibacillus plantarum* CRA52 to utilise whey permeate as a carbon source and to generate plantaricin and lactic acid with a yield of 543.48 AU/mL and 17.69 g/L, respectively [207]. Furthermore, *Lacticaseibacillus rhamnosus* can be a promising species for lactic acid production. Indeed, replacing glucose with lactose obtained from whey cheese in MRS medium did not affect the production of lactic acid. The highest concentration of lactic acid production was 27.5 g/L, and the cost of production was estimated to be about 0.11 € for one gram of lactic acid [208]. Dark fermentation, operated in a repeated-batch mode to mimic real-scale feeding strategies, was investigated for semi-continuous lactic acid production using mixed microbial cultures. Findings showed a maximum lactic acid concentration of 20.1 g/L and a maximum yield of 0.37 g of lactic acid per g of fed COD, both achieved with a hydraulic retention time of 2 days. Furthermore, it was reported that the generation of other products, including ethanol, acetic acid, and hydrogen, at concentrations of 5.5 g/L, 2 g/L, and 103 mL, respectively, occurred within 20 days of fermentation (phase 1 of dark fermentation: days 0–20) [209]. In fed-batch fermentation, *Lactobacillus delbrueckii* subsp. *bulgaricus* CGMCC 1.6970 produced 113.18 g/L of D-lactic acid from hydrolysed whey powder, with an average productivity of 2.36 g/L/h [210]. (ii) Succinic acid can be produced by anaerobic fermentation from whey from the rejects of milk-processing units using *Actinobacillus succinogenes* ATCC 55618. The productivity of succinic acid reached its maximum in 35 g/L of whey, accounting for 0.81 g/L/h, and yielded 62.1% [211]. Furthermore, cell immobilisation using alginate can increase succinic acid production. It was found that immobilising agents have improved the succinic acid yield to 74.9% by *Actinobacillus succinogenes* CCUG 43843T, with a productivity of 1.09 g/L/h achieved using cheese whey [212]. Further study investigated the potential of *Actinobacillus succinogenes* CCUG 43843T in producing succinic acid from delactosed whey permeate. The resulting succinic acid yield ranged between 0.62 and 0.67 g/g, which was compatible with commercial media containing glucose or lactose [213].

### 7.2. Polymers

Bio-based plastics have been found to be a good alternative to petroleum-based plastics. The production of polymers by *Paracoccus homiensis* using cheese whey was investigated. Results showed the ability of the selected strain to convert dairy processing residues into biopolymers, reaching a maximum production of 1.1 g/L and 0.8 g/L of biopolymer with cheese whey mother liquor and cheese whey, respectively, at 72 h [214]. Polyhydroxyalkanoates are biodegradable polymers that can be obtained through microbial fermentation of dairy residues. Scotta whey derived from ricotta cheese can be a promising substrate for batch fermentation with *Leuconostoc mesenteroides,* and after 24 h, the concentration of polyhydroxyalkanoates reached 0.09 g/L [215]. Likewise, it was reported that the ability of *Bacillus megaterium* NCIM 5472 to produce polyhydroxyalkanoate from whole cheese whey, with an accumulation of 75.7% of its dry weight and a yield of about 8.3 g/L [216]. A recent study investigated the use of cheese whey as a nutrient source for the diatom *Phaeodactylum tricornutum* in order to increase the fucoxanthin production yield. The addition of 3.3% of cheese whey to the new medium has enhanced the growth rate and increased fucoxanthin yield to 3.64 mg/L [217]. Further study investigated the production of oil from liquid cheese whey permeate using the yeast *Cutaneotrichosporon oleaginosus* through a two-step fermentation process. Yeast lipids were produced at a final concentration and productivity of 38 g/L and 0.57 g/L/h, respectively [218].

Bacterial cellulose has been gaining considerable attention in various sectors due to its biocompatibility, biodegradability, high water-holding capacity, and non-toxic properties [219]. It can be produced by aerobic bacteria, including *Aerobacter*, *Agrobacterium*, *Rhizobium*, *Azotobacter*, *Gluconacetobacter*, *Pseudomonas*, *Alcaligenes*, *Sarcina*, and *Rhodobacter* [220,221,222]. In this sense, Rollini and her colleagues have investigated the production of bacterial cellulose and Sakacin-A from cheese whey permeate, aiming to utilise it in antimicrobial packaging materials. The acetic acid bacterium *Komagataeibacter xylinus* DSM 2325 produced cellulose at a yield of about 6.8 g/L, which was later conjugated with sakacin-A produced by *Lactobacillus sakei* in a cheese whey permeate broth. An in vitro study demonstrated the efficacy of the obtained antimicrobial food package against the *Listeria* population on a fresh Italian soft cheese [223]. In addition, it is possible to produce polymers chemically from whey proteins by methacrylation and polymerisation reactions [224]. *Lactiplantibacillus plantarum* JNULCC001 showed an ability to convert cheese whey into functional exopolysaccharides [225]. The optimal condition was at 37 °C, pH 4.6, with a yield of 0.274 mg/mL. The obtained exopolysaccharide consisted of glucose and galactose units, linked by β1-4/6 bonds [226]. After 3 days of cultivation, the concentration of exopolysaccharides produced by *Enterobacter* sp. DSM 23139 reached 6.4 g/L, and its structure, contrary to that of *Lactiplantibacillus plantarum* JNULCC001 exopolysaccharide, was mainly composed of glucuronic acid and fucose [227]. *Leuconostoc mesenteroides* SJC113 was capable of producing mucoid exopolysaccharide with high β-galactosidase activity [228]. Exopolysaccharides production on whey permeate medium at 25 °C reached 477 mg/L in batch cultures with *Lactobacillus rhamnosus* RW-9595M. It was found that yeast nitrogen base supplementation has increased the production of exopolysaccharides [229].

### 7.3. Ethanol

Whey can be anaerobically digested into valuable compounds in biorefineries, such as methane and ethanol. In this regard, *Kluyveromyces marxianus* strains were capable of efficiently fermenting the lactose present in whey permeate into ethanol, achieving a yield of 90% of the theoretical maximum in the best case, while maintaining 90% cell viability [230]. In fact, some yeasts are capable of directly metabolising lactose whey. Among them, *Kluyveromyces fragilis*, *Kluyveromyces marxianus*, and *Candida pseudotropicalis* can ferment lactose directly using β-galactosidase and lactose permease [231]. Two yeast strains of *Kluyveromyces marxianus* and one strain of *Kluyveromyces lactis*, isolated from dairy products, were evaluated for 2-phenylethanol production on whey permeate-based media in batch cultures [232]. The use of *Kluyveromyces marxianus* resulted in biomass production and ethanol generation to about 75 g/L [233]. Batch fermentations of cheese whey permeate by *Kluyveromyces lactis* CBS2359 were performed in flasks with or without agitation to select the best conditions to produce simultaneously ethanol and biomass with high β-galactosidase activity. Findings revealed that the use of a twice-concentrated permeate in static culture was the optimal condition to maximise both ethanol concentration (22.2 g/L after 72 h) and β-gal activity (2083.0–2594.4 U/g over the whole fermentation), whereas the highest biomass content (3.25 g/L) was obtained in the same permeate using shake flasks [234]. Furthermore, cheese whey valorisation through ethanol fermentation using *Kluyveromyces marxianus* DSM 7239 has reduced costs associated with equipment and the overall process. Further improvement was observed when higher substrate concentrations were combined with a higher ethanol yield on lactose (0.45 g/g), leading to an initial drop in the Minimum Ethanol Selling Price (MESP) from 2.57 €/kg to 1.43 €/kg [235]. Nevertheless, *Saccharomyces cerevisiae* cells cannot ferment lactose because they lack β-galactosidase activity; therefore, a pretreatment with acid or enzymatic hydrolysis is required. As an example, the conversion of cheese whey into ethanol was achieved by the combination of both *Gluconobacter oxydans* and *Saccharomyces cerevisiae* cells. First, *Gluconobacter oxydans* partially oxidised the glucose, and the remaining galactose was further utilised for ethanol fermentation by *Saccharomyces cerevisiae*. As a result, one kg of cheese whey powder generates 290 g of gluconic acid and 100 g of ethanol [236]. Developing a system for direct lactose-to-ethanol fermentation leads to the creation of recombinant strains of *Saccharomyces cerevisiae* with lactose-metabolising genes. A mutant strain from *Saccharomyces cerevisiae* AY-51024M strain increased the lactose uptake rate to 1.041 g/L/h [237]. The metabolically engineered *Escherichia coli* W-pL13 strain demonstrated an efficient ability to ferment permeate and concentrated permeate without nutritional supplements, producing up to 40.5 g/L of ethanol from an initial lactose concentration of 117.4 g/L [238]. Additionally, mixed cultures have demonstrated an enhancement of bioethanol production. The co-culture of *Kluyveromyces marxianus* and *Saccharomyces cerevisiae* in shake flasks resulted in the consumption of 92% of total lactose and the production of 80–82 g/L ethanol, with an ethanol yield of approximately 86% [239]. It is well established that *Neolentinus lepideus*, a basidiomycete brown rot fungus, can produce ethanol not only from xylose but also from hexoses, including glucose, galactose, and lactose. The strain *N. lepideus* RS 1911 was capable of utilising lactose from cheese whey and expired cow’s milk, and generated 33 g/L ethanol after 192 h of fermentation (Initial lactose concentration 108 g/L) [240].

### 7.4. Methane

Recently, whey was regarded as an interesting substrate for methane and Hydrogen production (H_2_). Cheese whey powder collected from the dairy industry was assessed for methane production in a mesophilic (30 °C) expanded granular sludge bed reactor. It was found that hydrogen production increased in line with methane production, and the maximum reached methane yield was 9.8 mL/g COD applied [241]. The anaerobic digestion of cheese whey can be carried out using anaerobic granular sludge. Ultrasound was used on a whey sample to increase methane yield, with results showing that methane production kinetics increased by 46% after three days of digestion [242]. Further studies have investigated the efficiency of lab-scale continuous stirred tank reactors in methane production using cheese whey as feed. Results showed that after 35 days, methane production reached a peak of 1384 mL/L, dominated by hydrogenotrophic methanogens [243]. The anaerobic digestion of crude whey cheese without nutrient supplementation using *Mucor indicus* CCUG 22424, an edible filamentous fungus, was investigated. The microbial bioprocessing resulted in 6.11 kg m^−3^ fungal biomass, containing 0.733 kg m^−3^ chitosan, and bio-methane production of 27.6 m^3^ [244].

### 7.5. Peptides

Cheese whey was successfully utilised as a raw material for the production of low-molecular-weight peptides. Enzymatic and/or microbial treatments can generate novel peptides with interesting properties, including antimicrobial, antioxidant or immunomodulatory properties [124,245]. In this sense, enzymatic hydrolysis of whey protein using Flavourzyme, followed by lactose hydrolysis with β-galactosidase, has generated small peptides and saccharides that were visible after reverse-phase high-performance liquid chromatography-mass spectrometry [246]. Trypsin hydrolysis of cheese whey at 52 °C and pH = 8.2 produced small peptides with antioxidant capacity and angiotensin-converting enzyme inhibition [247]. Likewise, Zapata Bustamante and colleagues investigated the optimal hydrolysis conditions for whey protein concentrates to produce antioxidant and angiotensin-converting enzyme (ACE) inhibitory hydrolysates. Findings revealed that to obtain a hydrolysate with antioxidant potential, an enzyme: substrate ratio of 1:20 (*w*/*w*) and hydrolysis times of 7 h for alcalase, 2 h for chymotrypsin, and 6 h for flavourzyme were optimal [248]. Whey protein hydrolysates generated by two enzyme preparations (papain and a microbial-derived alternative) expressed high oxygen radical absorbance capacity, and inhibited dipeptidyl peptidase IV implicated in the regulation of type-2 diabetes [249]. Furthermore, it was reported that the generation of antibacterial peptides from crude cheese whey after pepsin and rennet enzyme hydrolysis, it was found that *Bacillus subtilis* and *Escherichia coli* were highly susceptible to pepsin and calf rennet digests, particularly pepsin prepared at pH 2.5–3.0 and calf rennet at pH 2.5–4.0 [250]. Additionally, it was reported that the α-glucosidase inhibitory activity of whey hydrolysates was obtained through further enzymatic degradation of rubbing cheese whey with papain at 46 °C for 240 min [251].

Whey protein hydrolysed by *Pediococcus acidilactici* SDL1414 showed a strong angiotensin 1-converting enzyme inhibitory activity of 84.7%, and mass spectrometry revealed that more than half (57.7%) of the obtained peptides in the *Pediococcus acidilactici* SDL1414 fermented samples were angiotensin-converting enzyme inhibitory peptides [252]. Further study evaluated the action of microbial proteolytic activity on whey proteins to release peptides with inhibitory activity of the angiotensin-converting enzyme. An increase in angiotensin-converting enzyme-inhibitory activity from 22% (unfermented whey) to 60–70% after 120 h of fermentation [253]. Olvera-Rosales and colleagues suggested co-culturing *Lacticaseibacillus rhamnosus* GG and *Streptococcus thermophilus* SY-102 to increase protein hydrolysis to 453 μg/mL (concentration of free amino groups), and the percentage of angiotensin-converting enzyme inhibition gradually increased from 21.4% to 52.4% by 21 h of fermentation [254]. It was reported that *Staphylococcus aureus* through lactoferrin expression [255]. Yeast-lactic fermentation of whey protein concentrate for 48 h with *Limosilactobacillus fermentum* and *Saccharomyces cerevisiae* has increased the availability of antioxidants, anti-hypertensive peptides, and enhanced anti-inflammatory response in macrophage cells [256]. Additionally, the co-culture of *Pediococcus pentosaceus* 147 with *Lactiplantibacillus plantarum* LE27 on a culture medium based on cheese whey has increased bacteriocin production, reaching 51,200 AU/mL [257].

Indeed, it was proposed to use cheese whey as an inducer for producing recombinant proteins due to its richness in lactose [10,258]. Recent studies have replaced traditional inducers with cheese whey permeate for the expression of ATX3-Q55, a toxic protein that can cause stress and cell damage when expressed in *Escherichia coli* cells. The growth curves of cells in the presence of cheese whey permeate showed an important growth, two times higher than the growth in the presence of Isopropyl β-d-thiogalactopyranoside (IPTG: a traditional inducer), and the glycerol was totally utilised in the first 24 h of induction. Regarding the amount of ATX3-Q55, it was 2 times higher in the presence of cheese whey permeate than with IPTG [259]. Likewise, cheese whey permeate and crude glycerol were utilised in a high-density fed-batch culture to produce recombinant β-galactosidase in *Escherichia coli* BL21 culture. Results revealed a 20% increase in cell biomass production after 48 h of incubation in cheese whey permeate, which may be attributed to the availability of micronutrients that alleviate cell stress. In a small-scale process (1.5 L fermentation), the entire production consumed 250 mL crude glycerol solution (equivalent to 125 g crude glycerol) and 300 mL cheese whey permeate (equivalent to 49.5 g lactose) to generate 52.9 g dry cell weight, 1980 kU recombinant soluble β-galactosidase, and 31.2 g/L galactose [260]. Nascimento and colleagues succeeded in producing β-galactosidase and mannosylerythritol lipids through direct conversion of cheese whey by *Moesziomyces antarcticus* PYCC 5048 and *Moesziomyces aphidis* PYCC 5535 [261]. Additionally, micro-filtrated cheese whey permeate was suggested as a sustainable substrate for the production of β-galactosidase by the yeast *Saccharomyces fragilis* IZ 275; after 12 h of incubation, only 11% of the substrate was left, and the biomass production was increasing over cultivation time, and the maximum yield of specific β-galactosidase activity 0.039 U/mg was obtained after 24 h of cultivation [262]. Further study aimed to optimise lipase production of *Bacillus subtilis* DSM 1088 using cheese whey as an eco-friendly substrate. Using Plackett-Burman and Central Composite Designs (PBD and CCD), whey, peptone, and agitation speed were identified as significant factors, achieving optimal lipase activity of 1314 U/mL, and the partial purification using ammonium sulfate (70%) resulted in a 2-fold increase in lipase activity, reaching 2628.2 U/mL [263]. *Meyerozyma guilliermondii* yeast was capable of producing acid lipase on cheese whey as the sole nutrient source, and under optimal conditions, the production yield reached 285.8 U/mL [264]. Indeed, *Escherichia coli* HMS174 cultivation using concentrated whey feed instead of a defined lactose feed resulted in 39% higher growth rates, 24% higher biomass yields, and even higher specific product titers for the model enzymes, flavanone 3-hydroxylase and chalcone 3-hydroxylase [265].

### 7.6. Other Metabolites (e.g., Vitamins, Glycerol, Volatile Fatty Acids)

Whey powder was used as a fermentative culture for the production of gamma-aminobutyric acid (GABA) using *Lactobacillus brevis* A3. The results of the central composite design of the response surface showed that lactic fermentation of 14.96% whey powder and 4.95% monosodium glutamate at a temperature of 37 °C and a fermentation time of 48 h generated the optimal GABA production, which was 553.5 ppm [266]. Further studies have shown the ability of *Lactiplantibacillus plantarum* and *Lactococcus lactis* subsp. *lactis* in co-culture, to produce 365 mg GABA per 100 mL of cheese whey- soy protein hydrolysate medium [267]. The strain *Enterococcus malodoratus* SJC25 converted glutamic acid to GABA in whey, and the GABA content in whey-based beverages reached 250–300 mg/100 mL [184]. Lactobionic acid production from ricotta cheese whey was measured after batch fermentation with *Pseudomonas taetrolens* strains. Fermentation and bioconversion yields of 34.25 ± 2.86 g/l and 85 ± 7% were achieved after 48 h. After 48 h of batch fermentation in a 3 L stirred tank bioreactor, the lactobionic acid titre reached 34.25 g/L, with a conversion yield of up to 85% [268]. *Klebsiella oxytoca* strain PDL-0 was further utilised to produce 2,3-butanediol from whey cheese. In fed-batch, recombinant strain *Klebsiella oxytoca* PDL-K5 produced 74.9 g/L 2,3-butanediol with a productivity of 2.27 g/L/h and a yield of 0.43 g/g from lactose [269].

Recently, several studies have investigated the production of vitamins, including B2, D, and A, using agro-industrial by-products as a low-cost substrate. In this sense, the production of carotenoids, a precursor of vitamin A, was achieved using the strain *Rhodotorula glutinis* P4M422, and after 72 h of incubation, the production reached its maximum, approximately 4075 µg/L carotenoids on goat whey milk medium [270]. Furthermore, the engineered strains of the yeast *Candida famata* produced 2.5 g/L of riboflavin with a yield exceeding 300 mg/g dry weight on whey supplemented with ammonium sulfate [271]. *Rhodotorula glutinis* was capable of generating 127.3 µg carotenoids from one gram of dry cheese whey hydrolysates. Whey hydrolysate was first treated enzymatically using β-galactosidase [272]. Further study affirmed the ability of *Kluyveromyces lactis* to generate phenolic compounds and short-chain fatty acids, including acetate, propionate, and butyrate, with concentrations 291.6 μg EAG/mL (Equivalents of Gallic Acid/mL), 8.8 mmol/L, 0.26 mmol/L, and 0.13 mmol/L, respectively, after fermentation of cheese whey-based beverage [28]. Cheese whey can also be an interesting substrate for enzyme production. Nattokinase production was proposed using cheese whey as low-cost fermentation medium. *Bacillus subtilis* produced nattokinase with a yield of 789.93 U/mL. It was found that supplementing cheese whey with yeast extract (10 g/L) can increase caseinolytic activity to 833.43 U/mL [273]. Additionally, the use of cheese whey for *Pleurotus djamor* growth can serve as an alternative to produce bioactive compounds, including ergosterol and β-glucans [274]. It was reported that enzymatic hydrolysis with the digestive enzymes of cheese whey can increase antioxidant and antihypertensive peptides, showing a maximum DPPH-scavenging activity of 26% [247].

## 8. Conclusions and Perspectives

Whey is transitioning from a high-load effluent to a flexible feedstock for foods and bio-based manufacturing. Scalable fractionation (UF/NF/ED) and bioprocessing unlock WPC/WPI, lactose-derived prebiotics, peptides, organic acids, ethanol, and polymers, while tailored fermentations enable functional beverages, bakery improvers, and clean-label preservation. Currently, whey and whey derivatives are widely used as gelling agents, foaming agents, and emulsifiers. In addition, promising approaches are being developed to exploit whey in the biomass production of several probiotic microbes, high-value compound production, food packaging polymer production, cosmetics formulation, biofuels, and as a delivery system for bioactive compounds. Key bottlenecks now lie in acid–whey palatability (mineral and lactic acid load), debittering of hydrolysates, and robust control of pathogens, spores, and chemical residues. The review prioritises (i) integrated demineralisation–deacidification trains, (ii) debittering by targeted proteolysis/encapsulation, (iii) standardised safety bio-tools (bioprotective cultures + non-thermal hurdles), and (iv) techno-economic and LCA benchmarks. Further studies are needed to ensure the combination of economic and environmental sustainability, with the possibility of integrating Artificial Intelligence to predict additional valorisation approaches and process optimisation.

## Figures and Tables

**Table 1:** The chemical composition of milk and whey based on milk origin, according to recent findings.

		Total Solids (% *w*/*w*)	Protein (% *w*/*w*)	Fat (% *w*/*w*)	Lactose (% *w*/*w*)	Ash (% *w*/*w*)	COD (g/L)	BOD (g/L)	Reference
**Milk**	Cow	11.8–13	3.0–3.9	1.3–5.4	4.2–5.6	0.6–0.8			[59,60,61]
	Buffalo	15.7–17.2	2.7–4.7	5.3–9.0	3.2–4.9	0.8–0.9			[60]
	Goat	11.9–16.3	2.5–5.2	3.0–7.2	3.2–5.0	0.7–0.9			[60,61,62,63]
	Sheep	18.1–20.0	4.5–7.0	5.0–9.0	4.1–5.9	0.8–1.0			[60]
	Human		1.1–1.4	3.5–3.8	6.6	0.1–0.2			[61,62]
**Whey**	Cow	6.6	0.8	0.2	5.0		50–102	27–60	[64,65]
	Buffalo	6.0–7.0	0.7–0.9	0.1–0.8	4.2–5.0				[10,37]
	Goat	5.1	0.43	1.2	4.1				[64]
	Sheep	9.5	1.75	1.5	3.7				[64]

Biochemical oxygen demand (BOD); Chemical oxygen demand (COD).

## Data Availability

No new data were created or analysed in this study.
